# Recent Updates on Source, Biosynthesis, and Therapeutic Potential of Natural Flavonoid Luteolin: A Review

**DOI:** 10.3390/metabo12111145

**Published:** 2022-11-20

**Authors:** Nandakumar Muruganathan, Anand Raj Dhanapal, Venkidasamy Baskar, Pandiyan Muthuramalingam, Dhivya Selvaraj, Husne Aara, Mohamed Zubair Shiek Abdullah, Iyyakkannu Sivanesan

**Affiliations:** 1 Department of Plant Pathology and Microbiology, The Robert H. Smith Faculty of Agriculture, Food and Environment, The Hebrew University of Jerusalem, Rehovot 76100, Israel; 2 Department of Biotechnology, Karpagam Academy of Higher Education, Coimbatore 641021, Tamil Nadu, India; 3 Centre for Plant Tissue Culture & Central Instrumentation Laboratory, Karpagam Academy of Higher Education, Coimbatore 641021, Tamil Nadu, India; 4 Department of Oral & Maxillofacial Surgery, Saveetha Dental College and Hospitals, Saveetha Institute of Medical and Technical Sciences (SIMATS), Saveetha University, Chennai 600077, Tamil Nadu, India; 5 Division of Horticultural Science, College of Agriculture and Life Sciences, Gyeongsang National University, Jinju 52725, Republic of Korea; 6 Department of Computer Science and Engineering CSE-AI, Amrita School of Engineering, Chennai 601103, Tamil Nadu, India; 7 Department of Bioresources and Food Science, Institute of Natural Science and Agriculture, Konkuk University, 1 Hwayang-dong, Gwangjin-gu, Seoul 05029, Republic of Korea

**Keywords:** anti-inflammatory, anti-cancer, anti-diabetic, luteolin, secondary metabolite, flavonoid

## Abstract

Nature gives immense resources that are beneficial to humankind. The natural compounds present in plants provide primary nutritional values to our diet. Apart from food, plants also provide chemical compounds with therapeutic values. The importance of these plant secondary metabolites is increasing due to more studies revealing their beneficial properties in treating and managing various diseases and their symptoms. Among them, flavonoids are crucial secondary metabolite compounds present in most plants. Of the reported 8000 flavonoid compounds, luteolin is an essential dietary compound. This review discusses the source of the essential flavonoid luteolin in various plants and its biosynthesis. Furthermore, the potential health benefits of luteolins such as anti-cancer, anti-microbial, anti-inflammatory, antioxidant, and anti-diabetic effects and their mechanisms are discussed in detail. The activity of luteolin and its derivatives are diverse, as they help to prevent and control many diseases and their life-threatening effects. This review will enhance the knowledge and recent findings regarding luteolin and its therapeutic effects, which are certainly useful in potentially utilizing this natural metabolite.

## 1. Introduction

Plants have a vast majority of chemical compounds which are used daily. Due to knowledge of plant-based therapeutic benefits, bioactive compounds have been explored in past decades to treat various human diseases in addition to being utilized in their prevention [1]. The uses of these chemical compounds in dietary and therapeutic applications significantly impact well-being, as most of these compounds have beneficial activities for healthy living. Among the phytochemical compounds, flavonoids are the major group due to their beneficial properties. The flavonoids are polyphenols having a C6-C3-C6 diphenylpropane structure and two benzene rings. As of today, more than 8000 flavonoid compounds have been identified and differentiated based on their heterocyclic C-ring structure into 10 groups, among which flavones, flavanones, chalcones, flavanols, isoflavones, and anthocyanins have pharmacological benefits [2,3]. In plants, their role is involved in protecting the plant cells against ultraviolet radiation and biotic stresses. They act as anti-microbial compounds, give color to the flowers, and thus help pollination [4,5].

**Figure 1:**
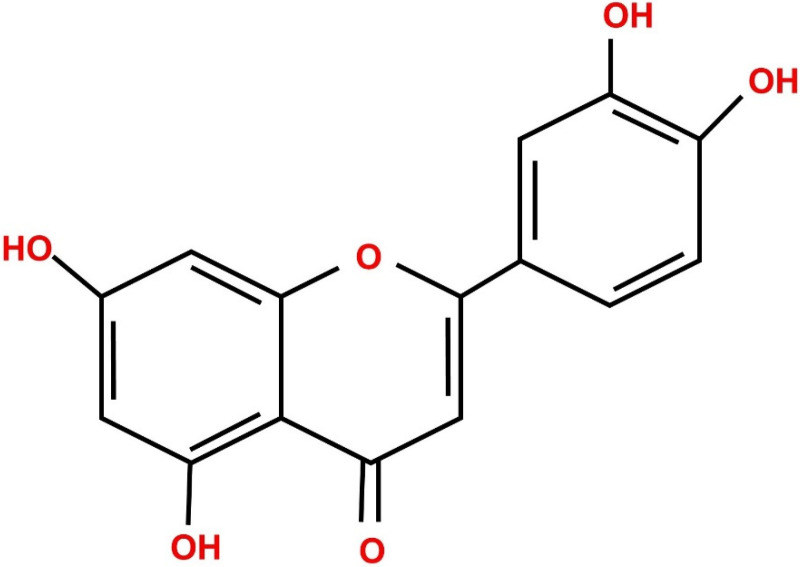
Diagrammatic representation of luteolin compound.

The flavonoid compounds structural activities depend on their hydroxyl groups. Among the flavonoids, luteolin (3′,4′,5,7-tetrahydroxy flavone) is an important dietary compound present in different plant species [6]. Most of the bioactivity of luteolin (LUT) is due to a hydroxyl moiety present in the position of 3′, 4′, 5, and 7 carbon (Figure 1). Luteolin is a widely present flavonoid compound, as its major source is fruits, vegetables, and other edible parts of plants. Research studies in the past decades explored the biological significance of the LUT compounds, revealing their antioxidant, anti-cancer, anti-inflammatory, and neuroprotective nature [7,8]. As these compounds therapeutic effects are increasing, this review will enlighten more on LUT for a deeper understanding in addition to highlighting current research.

## 2. Source of Luteolin

**Figure 2:**
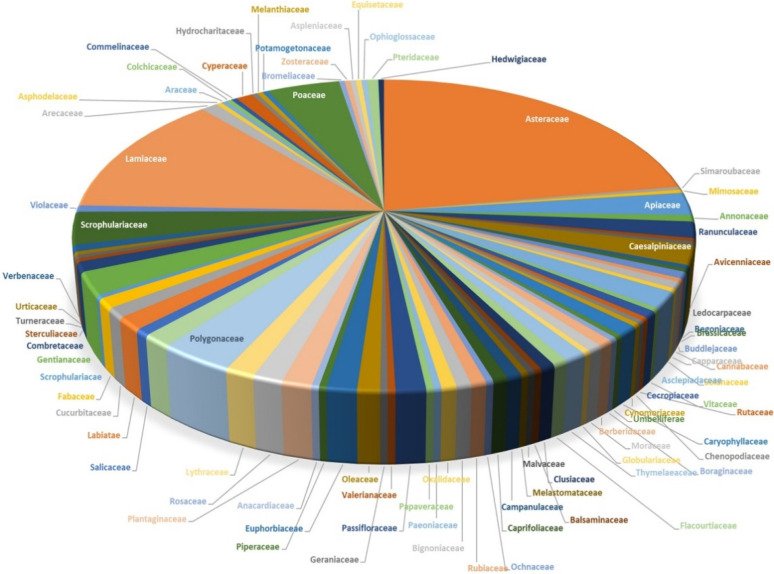
Distribution of luteolin compounds in the plant kingdom. The presence of luteolin in various plant families is represented in the pie chart based on the number of species in each family.

Luteolin’s therapeutic benefits have led the scientific community to explore its potential more. The search in the NCBI PubMed database retrieved more than five thousand articles showing its potential nature. The plant kingdom is the major source of this compound, and it is present as LUT or as luteolin glycosides. Its wide distribution among plants is well documented, as more than 300 plant species were reported to possess LUT or its derivates [9]. Its presence is even documented in the 36- and 25-million-year-old fossils of *Celtis* and *Ulmus* species, respectively [10]. The presence of LUT was identified among monocotyledons and dicotyledons. Among the plant kingdom, in the families of Asteraceae, Lamiaceae, Poaceae, Leguminosae, and Scrophulariaceae species, the LUT and its glycosides were identified in 66, 38, 13, 10, and 10 species, respectively (Figure 2).

## 3. Luteolin Biosynthesis

Plants have an array of metabolic compounds for their basic functions and their response to various stimuli. Phenyl propanoids are compounds involved in plant development, cellular metabolism, and biotic and abiotic stimuli, and are obtained from the phenylalanine molecule [11]. The phenylpropanoid pathway starts after the shikimate pathway that has been studied for decades [12]. The LUT molecule is the product of the phenylpropanoid and flavonoid pathways, as it branches from the major secondary metabolite pathway, the phenylpropanoid pathway, where all the secondary metabolic compounds are synthesized.

**Figure 3:**
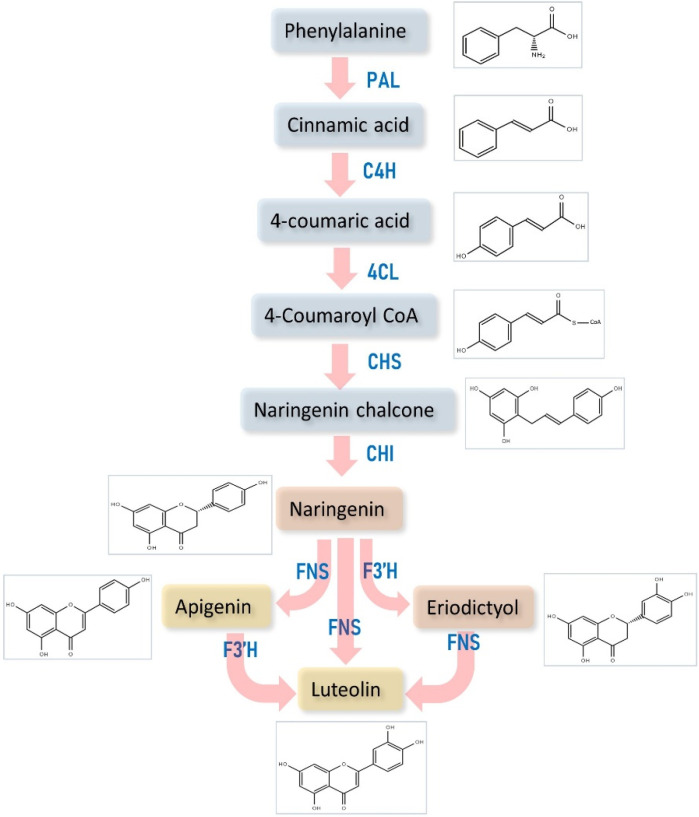
Diagrammatic representation of the biosynthesis of luteolin. PAL—phenylalanine ammonia lyase; C4H—cinnamate 4-hydroxylase; 4-CL—coumarate 4-ligase; CHS—chalcone synthase; F3′H—flavonoid 3′-hydroxylase; CHI—chalcone isomerase; FNS—flavone synthase.

The flavonoid biosynthesis starts with phenylalanine ammonia lyase converting phenylalanine (Phe) amino acid into trans-cinnamic acid, followed by trans-coumaric acid using enzyme trans-cinnamate 4-hydroxylase (C4H), p-coumaroyl CoA by enzyme coumarate 4-ligase (4CL) [11,13], which is then converted into naringenin chalcone (NC) by the enzyme chalcone synthase, a type III polyketide synthase family enzyme [14]. NC is transformed into naringenin, an important compound acting as a key step in the luteolin biosynthesis by the enzyme chalcone isomerase (CHI) [15]. Further, naringenin is converted into eriodictyol by the enzyme flavonoid 3′-hydroxylase (F3′H), as this enzyme introduces a hydroxyl group at the 3′ position in the beta ring [16]. In addition, flavone synthase (FNS) belongs to the cytochrome P450 superfamily, which produces LUT from the substrate naringenin and eriodictyol [17,18] (Figure 3).

## 4. Physiochemical Properties of Luteolin

The luteolin molecule in plants is widely distributed as the aglycone molecule without a sugar moiety and as a glycoside molecule with a sugar moiety bound to it. Its molecular formula is C_15_H_10_O_6_ with an MW of 286.24 [19]. Luteolin has weak aqueous soluble properties [20]. Most of the LUT molecule occurs as O-glycosides, having aglycone attached with sugar moieties by one or more hydroxyl (OH) group. The OH groups are positioned at the 5, 7, 3′, and 4′ position. Among the sugar moieties, glucose is the major sugar molecule attached to luteolin. Other than that, rhamnose, rutinose, arabinose, xylose, and glucuronic acid are other sugar derivatives attached to luteolin [9,21].

## 5. Chemopreventive Functions of Luteolin

Luteolin is one of the natural secondary metabolites derived from plants shown to possess various chemopreventive activities such as antioxidant, anti-cancer, anti-microbial, anti-diabetic, anti-inflammatory, and neuroprotective functions.

### 5.1. Antioxidant Properties of Luteolin

Oxidative stress plays a major role in various cellular metabolisms and also during the pathogenesis of neurodegenerative disorders, cancer, diabetes, cardiovascular diseases, rheumatoid arthritis, aging, and hypertension [22]. Oxidative stress occurs due to the production of reactive oxygen species (ROS) formed during the oxidative phosphorylation of oxygen to produce energy by synthesizing ATP. There are different ROS molecules present, such as hydroxyl radical (OH), hydrogen peroxide (H_2_O_2_), peroxynitrate (ONOO^−^), and superoxide anion (O_2_^−^). The balanced production of antioxidant molecules maintains balanced cellular homeostasis, whereas an imbalanced mechanism of overproduction of ROS leads to oxidative stress. Thus, the antioxidative molecules help protect from developing oxidative stress in various diseases.

Flavonoids have antioxidant properties that have been widely reported in several research findings [23,24]. The LUT molecule among the flavonoids has antioxidant activity, as it possesses anti-scavenging activity due to its glycosidic group, which helps in removing reactive nitrogen species and oxygen species [25,26,27,28,29]. LUT’s antioxidant activity is linked to the C-glycosylation effect at various positions, which causes the intensity and changes in its scavenging properties [30]. The luteolin (50 mg/kg orally) pretreatment gives protection against renal failure through the detoxification mechanism by antioxidant activity and anti-inflammatory and anti-apoptotic mechanisms in Wister rats [31]. In addition, LUT helps in reducing the effect of mucosal damage due to intestinal mucositis caused during cancer treatment [32]. The hepatoxicity induced by carbon tetra chloride (CCl_4_) in the rat model was reduced by LUT’s antioxidant property by increasing the activity of various antioxidant enzymes [33].

Furthermore, the LUT antioxidant activity was proved to induce apoptosis via increasing antioxidant activity [34]. LUT from *Reseda odorata* L. reduces severe acute pancreatitis (SAP) by activating hemeoxygenase-1 (HO^−1^)-based anti-inflammatory and antioxidant activity via suppressing nuclear factor-κB (NF-κB) [35]. LUT also acts as a chemoprotective molecule during doxorubicin treatment which causes hepatorenal injuries, as it helps in the therapeutic efficiency of the drug by removing its toxic effect due to its antioxidant nature [36]. Thus, the effective role of the LUT molecule and its glycosides mediates a crucial action in various metabolic processes and acts as a protective molecule by reducing the ROS species through its antioxidant activity.

### 5.2. Anti-Cancer Activity

**Figure 4:**
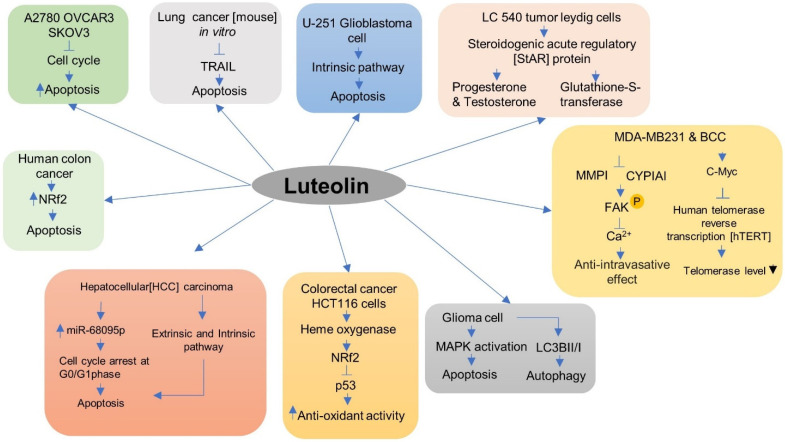
The anti-cancer effect of luteolin compounds against different cancer cells was sketched. The anti-cancer activity of the compound and its mechanism for preventing cancer cells involving different mechanisms.

Cancer is the deadliest disease affecting human beings, as the global death ratio is ever increasing due to its uncurable nature. As it alone causes around 10 million deaths, which implies that one in every six people dies from it [37]. The most common cancers are lung, breast, colon, prostate, stomach, liver, ovary, thyroid, and rectum. The cause of the higher emergence of cancers is due to lifestyle changes such increased tobacco and alcohol consumption, enhanced body weight index, lack of physical exercise, and lower dietary food intake. Adjoining diseases such as human papillomavirus (HPV) cause nearly 30% of death due to cancer [38,39,40]. The luteolin molecule has anti-cancer and anti-inflammatory properties [41] (Figure 4 and Table 1). An in silico analysis of the LUT molecule from *Tridax procumbens* showed it to have high active probability and less cardio-toxicity, making it an ideal drug candidate targeting the mini chromosome maintenance (MCM7) protein which causes dysregulation of DNA and leads to various types of cancer [42]. 

The LUT molecule has anti-cancer properties attributed to its antioxidant and free radical quenching activity [41]. Its effective inhibitor activity against cancer cell proliferation studied both in vivo at a dosage of 3 to 50 µM and in vitro at a dosage of 5 to 10 mg/kg proved its efficiency [43]. Its ability to penetrate the skin gives the advantage of treating skin cancer. Studies involving human carcinoma cells have shown its activity against stomach cancer at an IC50 value of 7.1 µg/mL. Against lung cancer, its effective activity was seen at an IC50 value of 11.7 µg/mL, and an IC50 value of 19.5 µg/mL was found to be effective against bladder cancer [44]. Blood cancer leukemia is another major cancer affecting humans, as it produces abnormal white blood cells, causing many deaths. The LUT compound also showed an inhibitory effect on the human leukemic cell lines CEM-C1 and CEM-C7 [45,46].

In addition, its growth inhibitory effects are evidenced in a study against HL-60, the human promyelocytic leukemia cells. LUT from the fruit of *Vitex rotundifolia* has a growth inhibitory concentration of 15 µM at 96 h [47,48]. Also, when STZ-induced diabetic rat models administered with luteolin it improves cognitive function, as it reduces in the diabetic condition. The improvement in expression of growth-associated protein-43 (GAP-43) and synaptophysin (SYN) in the hippocampus after LUT treatment was also found [49].

**Table 1:** The mechanistic action of luteolin and its types against various types of cancer cells.

Compound	Cancer Cell	Mechanism	Reference
Luteolin	Colorectal cancer HCT116 cells	It increases the transcriptional activity of antioxidative response element in HCT116 cells.	[50]
Luteolin-7-O-glucoside and luteolin	MCF-7 cell in breast cancer	Anti-cancer activities against MC77 cells with selective index 8.0.	[51]
Apigenin and luteolin	MDA-MB231 breast cancer cells (BCC) immortalized lymph endothelial cell (LEC) monolayer	It suppresses pro intravasation trigger factors in MDA-MB 231 BCC, specifically MMP1 expression and CYP1A1 activity.	[52]
Luteolin	A2780, OVCAR3, and SKOV3	By inducing apoptosis, arrested cell cycle thus inhibits cell invasion in ovarian cancer cells.	[53]
Luteolin	Myeloid leukemia cells	It triggers leukemia cells apoptosis through modulating the differential expression of PTTG1.	[54]
Luteolin	Lung cancer (mouse) in vitro	It enhances inhibition of tumor growth, thus decreases tumor weight and increases tumor cell apoptosis in vitro.	[33]
Luteolin	Tumor cells	It reduces the tumorigenic potential and inhibits the migration of U-251 glioblastoma cells. It enhances apoptosis by an intrinsic pathway.	[55]
Nano Luteolin	Lung cancer (H292 cell) and head and neck cancer (SSCH and TU212) cell line	Nanoluteolin inhibits the effect of tumor growth of SCCHN.	[56]
Luteolin	Hepatocellular (HCC) carcinoma	It represses the growth of HCC by stimulating apoptosis and cell cycle arrest at G0/G1phase in Huh7 cells at the G2/M phase; miR-68095p mediates the growth-repressive activity of luteolin in HCC.	[57]
Luteolin	Colon cancer cells	It induces apoptosis in doxorubicin-sensitive LoVo colon cancer cells and drug-resistant LoVo/Dx cell lines. Their cytotoxic activity in LoVo/Dx cell line was considerably lower than LoVo cell line.	[58]
Luetolin-7-O-glucoside	Nasopharyngeal carcinoma (NPC-039 NPC-BM)	It reduces the proliferation of NPC cell line by inducing S and G2/M cell cycle arrest by chromatin condensation at apoptosis through AKT signaling pathway.	[59]
Luteolin	4TI breast cancer cell	It increases the apoptosis in 4TI BCC.	[60]
Luteolin	Breast cancer cell MDA-MB231	It reduces telomerase levels in a concentration-based fashion. It inhibits phosphorylation of the NF-κB inhibitor and its target gene c-Myc to repress human telomerase reverse transcription (hTERT) expression that codes the catalytic subunit of telomerase.	[61]
Luteolin	Tamoxifen resistant ER (TRER) + VE Breast cancer cells	The synergistic application of luteolin and P13K, AKT, or mTOR inhibitors synergistically enhances apoptosis in TRER+VE cells. Ras gene (*K-Ras*, *H-Ras*, and *N-Ras*) inducer of P13K was transcriptionally suppressed by stimulation of tumor suppressor mixed-PI3K lineage leukemia 3 (MLL3) expression.	[62]
Luteolin	Hepatocellular cancer Hep 3B cells	It induced autophagy in p53 null Hep3B cells.	[63]
Luteolin	Human colon cancer	It inhibits the expression of DNA methyltransferase, a transcription repressor that enhanced the expression of the activity of ten-eleven translocation (TET) DNA methylase a transcription activator. It also increases the interaction between Nrf2 and p53, which increases the expression of antioxidative enzymes and apoptosis-related protein.	[34]
Luteolin	Glioma cell	It inhibits glioma cell proliferation in a time- and concentration-based fashion by glioma cell apoptosis via MAPK induction (JNK, ERK, and P38) and autophagy	[64]
Luteolin	LC 540 tumor Leydig cells	It activates steroidogenic acute regulatory (StAR) protein expression and increases progesterone and testosterone production. It also controls the expression of genes that participate in stress responses such as glutathione-S-transferases Gsta1 and Gstt2 and the unfolded protein response.	[65]
Luteolin	Amelanotic melanoma C32 (CRL-1585) cells	Luteolin and its derivatives demonstrate significant cytotoxic and pro-apoptotic potential.	[66]
Luteolin-7-O-glucoside	Oral squamous cell carcinoma	It reduces the oral cancer cell migration and invasion, causing a decrement in cancer metastasis by decreasing p38 phosphorylation by reducing matrix metalloproteinase (MMP)-2 expression. It exerts an anti-migratory effect by inhibiting P38-induced enhanced expression of MMP-2 and also by the extracellular signal regulatory kinase pathway.	[67]

### 5.3. Anti-Diabetic Activity

Diabetes is a significant health concern worldwide. Its prevalence is felt in every developed and developing country. Nearly 451 million people are affected by it, according to the 2017 International Diabetic Federation (IDF) report, which projects a further increase to 693 million by 2045. It also has severe socio-economic effects. Diabetes among the younger population is increasing, which alarms society. Diabetes is among the top diseases that affect the world population’s health and lead to various life-threatening illnesses [68]. LUT, a secondary plant metabolite, has anti-diabetic properties, as established in multiple studies. On administration, it lowers the seizure threshold due to its antiepileptic activity. 

In addition, its neuroprotective property helps reduce the kainic-acid-induced neuronal cell death in the hippocampal CA3 region. When used as pretreatment, it protects the morphological appearance of the nerve cells’ nucleus, mitochondria, and endoplasmic reticulum, while restoring the ultrastructure of the nerve cells [69]. Diabetes affects the heart muscles and causes myocardial I/R or damage due to oxidative stress. Upon treatment with LUT, oxidative stress and damage to the heart are reduced by the redirection in the oxidation reaction via activating the sestrin 2-Nrf2-based feedback loop [70]. 

Long-term diabetes impacts the cerebral cortex neurons; the administration of luteolin significantly reduces diabetic conditions, including lipid peroxidation, as it increases in diabetic rat brains and also reduces GS4, superoxide dismutase, and catalase activity, which markedly decrease in the cerebral cortex and hippocampus of rats upon administering luteolin. This implies that luteolin’s antioxidant action helps improve CA1 neurons by reducing neuronal apoptosis, as ChE activity results from diabetes, leading to progressive cognitive impairment and neurological dysfunction. In treating diabetic rats with LUT, the ChE activity is inhibited, thus improving the condition [71]. 

An in silico molecular docking study showed that LUT binds to alpha-amylase and dipeptidyl peptidase IV (DPP IV) efficiently. Thus, it prevents glucose optimization and then binding to glutamine-fructose-6-phosphate amido transferase (GFAT1), and Forkhead box protein O1 (FOX01), suggesting that it may help to avoid hyperglycemia. This shows that LUT is a potent inhibitor of type 2 diabetes mellitus [72]. During kidney hemorrhage, LUT significantly decreases MDA levels and increases SOD activity. It also restores the enhanced level of serum lipids in diabetes mellitus, as the increased level leads to diabetic nephropathy. Its antioxidant properties help to decrease oxidative stress by stabilizing the membrane lipids and thus reducing oxidative damage. Luteolin’s renoprotective effects relate to enhancing HO-1 expression and inducing antioxidants in diabetic nephropathy. Luteolin prevents the morphological damage of the kidney caused by diabetes mellitus [21]. However, intense research is warranted to examine the mechanism of luteolin’s renoprotective effects.

In another study, compared to untreated cells, LUT significantly increased PI3K and IRS1/2 expression in a dose-dependent manner. These findings demonstrate that in the adipocytes IRSI 1/2 and PI3K pathway-dependent insulin sensitivity was seen. The fact that LUT prevented p65 from moving from the cytosol to the nucleus suggests that it reduces adipocyte inflammation by preventing NK-кB cell activation [73]. 

Luteolin is a non-competitive inhibitor of alpha-glucosidase, as it binds to enzymes, whether at low or high concentrations. It suggests that LUT has the strongest affinity for alpha-glucosidase enzymes and BACE1 [74]. Luteolin acts as a potent, highly effective, non-competitive reversible inhibitor of alpha-glucosidase [75]. Due to its low IC50, LUT exhibits the strongest dipeptidyl peptidase IV (DPP IV) inhibitory activity. A kinetic study revealed that LUT inhibits DPP IV in a non-competitive manner and binds to the S3 and S2 proteins. The side chain of amino acid residues may change in DPP IV confirmation due to S2 and S3 binding. The IC of DPP IV is required to inhibit 50% of enzyme activity [76].

### 5.4. Anti-Inflammatory Activities

**Figure 5:**
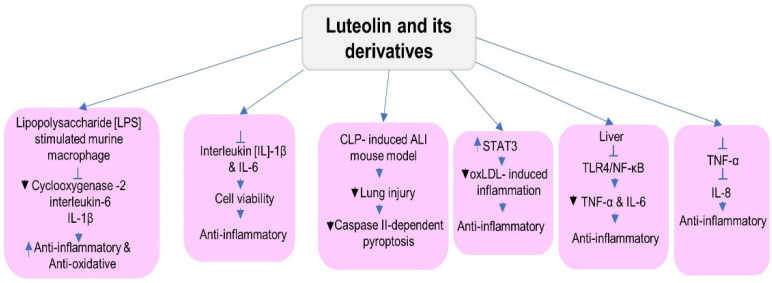
The figure representing the anti-inflammatory mechanism of luteolin in various cells.

Inflammation is a response to stimuli induced by immune cells and non-immune cells in our body by involving various biochemical pathways and different molecules. It is a natural process of how the body responds to a stimulus with the help of immune cells such as natural killer cells, macrophages, and their molecular pathways. However, the inflammation response is needed to reduce the impact of the stimuli, further affecting the normal cells, but prolonged inflammation affects normal functioning as it leads to chronic conditions, so it needs to be prevented. For managing it, anti-inflammatory molecules are administered to protect cells from adverse effects [77]. Luteolin has anti-inflammatory properties, which are shown in Figure 5. Luteolin decreases the oxLDL-activated inflammation by inhibiting a signal transducer and activator of transcription 3 (STAT3) in vitro. One study showed its interaction with STAT3 primarily through hydrogen bonds [78]. Luteolin administration alleviates lung injury by attenuating caspase-2-based pyroptosis in the lung tissue of cecal ligation and puncture (CLP). Also in the induced ALI mouse model, it regulates the mechanism related to the frequency of regulated T cells (Treg) and the Treg-derived IL-10 [79]. 

Luteolin-7-O-glucuronide [L7Gn] revealed anti-inflammatory and antioxidative properties in lipopolysaccharide (LPS)-stimulated murine macrophages, as the mRNA expression of inflammatory mediators including cyclooxygenase-2 (COX-2), interleukin-6, and IL-1B was inhibited by luteolin-7-O-glucuronide treatment [80]. The co-system of LUT and quercetin was analyzed as the combination of repressed TNF-α production and IL-8 mRNA expression, thus indicating anti-inflammatory and anti-allergic activities [81]. Also in another study, Luteolin-7-O-glucoside (LUT-7G) prevents damage to cardiovascular tissues by lowering the generation of ROS is established by the inhibition of STAT3 and the downregulation of target genes involved in inflammation [82]. Luteolin could inhibit the TLR4/NF-κB pathway, thus reducing the inflammatory factor TNF-α and IL-6 in plasma, liver, and ileum to reduce liver inflammation [83].

Matrix metalloproteinase 9 (MMP-9) plays a critical role in the inflammatory response. One study established that LUT decreases MMP-9 expression to treat ischemic stroke, colon cancer, and diabetes. CASP3 is the main terminal-cleaving enzyme, and the activation of CASP3 causes apoptosis and inflammation, but LUT can increase CASP3 expression to induce apoptosis in the HaCat cells and cancer cells [84]. Luteolin significantly improved the caerulein plus LPS induced in severe acute pancreatitis (SAP) mice. Increased HO-1 levels decreased NF-кB activity and increased anti-inflammatory activity [35]. Further, LUT is reported to process anti-inflammatory activity with the mechanism of having COX-2, interleukin, and TNF as molecular targets. Luteolin-7-0-β-D-glucuronide inhibited the NO and pro-inflammatory cytokine production [85]. The research shows that TNF-α induced a considerable reduction in HNPC (human nucleus pulposus cell) viability and an increase in inflammatory factor levels. In contrast, application with LUT shows enhanced cell viability and reduced intracellular interleukin (IL)-1β and IL-6 expression levels [86]. 

Luteolin also reversed TNF-α-induced senescence and suppressed TNF-α, causing inflammatory injury. In addition, luteolin-3′-O-phosphate (LTP) shows better anti-inflammatory activity by inhibiting the mitogen-activated protein kinase and NF-κB more effectively than luteolin. Also, at the concentration of 10 µM, LTP showed higher anti-inflammatory activity in comparison to luteolin [87]. In a previous study, it was found that LUT caused in vitro activation of NF-κB and AP-1. However, LUT exhibited a more potent anti-inflammatory activity than luteolin-7-O-glucoside in Ga1N/LPS-intoxicated ICR mice [88]. The STAT3 pathway is the potential target of LUT, which reduces renal fibrosis and delays the progress of diabetic nephropathy [89]. 

### 5.5. Protection against Alzheimer’s Disease

Alzheimer’s disease is a prime cause of memory loss among the world’s population. The main characteristic of the disease is the accumulation of β amyloid peptides in the brain’s extracellular matrix [90]. A treatment to prevent the condition has not been established. Still, research is ongoing around the globe to find the cure for Alzheimer’s disease. In this view, the secondary metabolite LUT has some potential to reduce the condition. LUT effectively reduces Alzheimer’s disease symptoms and the formation of Aβ42 aggregation in transgenic drosophila due to the (direct) interaction of ROS with the gene expression of an antioxidant enzyme involved in free radical scavenging. This is shown via a reduction in AchE activity in a concentration-mediated manner, which results in the slowing down of the inception of Alzheimer’s disease-like symptoms [91]. Luteolin improves brain histomorphology and decreases protein plaques in 3XTg-Alzheimer’s disease mice, as it inhibits neuro-inflammatory aggravation by repressing ER stress, which causes learning and memory impairment in mice [92].

Further, it significantly reduced the expression of Bax and caspase-3 and induced the expression of Bcl2. A high amount of LUT may have potential toxicity, inhibiting Aβ25-35 and inducing cell apoptosis. It also activates the ER/ERK/MAPK signaling pathway to protect Bcl2 cells against Aβ25-35 and induce apoptosis via specifically acting on ERβ [93]. Luteolin may decrease brain insulin resistance. The present studies found that the LUT treatment potentiated insulin signaling in the hippocampus and increased glucose metabolism by increasing hepatic insulin sensitivity and the tight regulation of β-cell function [94].

### 5.6. Luteolin in Parkinson’s Disease (PD) Treatment

Luteolin produced during counteraction in the in vitro effect on oxidation is associated with the abnormal enhancement of endogenous free radical suppression of the mitochondrial viability of mitochondria membrane potential and a decrease in the glutathione content. The catalyzing activity indicates that the multilayer modulatory pathway plays a role in luteolin neuroprotection activity. The protection is a result of possible balancing in the pro-oxidation or antioxidation ratio. Further, the neuroprotective mechanism helps to restore the depressed endogenous enzymatic and non-enzymatic antioxidative defense system known as ROS scavenging activity [95]. Luteolin-7-O-glucoside helps to protect against dopaminergic neuro injury in the SH-SY5Y human dopaminergic neuronal cell line, where it increased the cell viability of a 1-methyl-4-phenylpyridinium iodide (MPP+)-treated SH-SY5Y cell line by suppressing apoptosis, as was visible by decreased nuclear condensation. 

Furthermore, it increases the Bcl2/Bax ratio by reducing caspase-3, and also prevents the depletion of TH+ve neurons in the substantia nigra (SN) and neuro fibers in the striatum, thus improving mice behavior in the pole trait and traction test and implicating its potential in applied PD therapy [96]. The cell viability is lost due to 6-OHDA-induced apoptosis in PC12 cells, during which the Bax/Bcl2 ratio is enhanced along with p53 expression. The 6-OHDA induces BIM and TRB3 mRNA expression, affecting cellular viability. Treating with the LUT inhibits 6-OHDA-induced apoptosis and blocks BIM and TRB3 mRNA expression, thus increasing cell viability loss. This indicates neuroprotective activity [97,98]. Further, the luteolin administration reduces the H_2_O_2_-induced cell apoptosis through the Bcl2 pathway, thus improving neuronal synaptic plasticity. 

**Figure 6:**
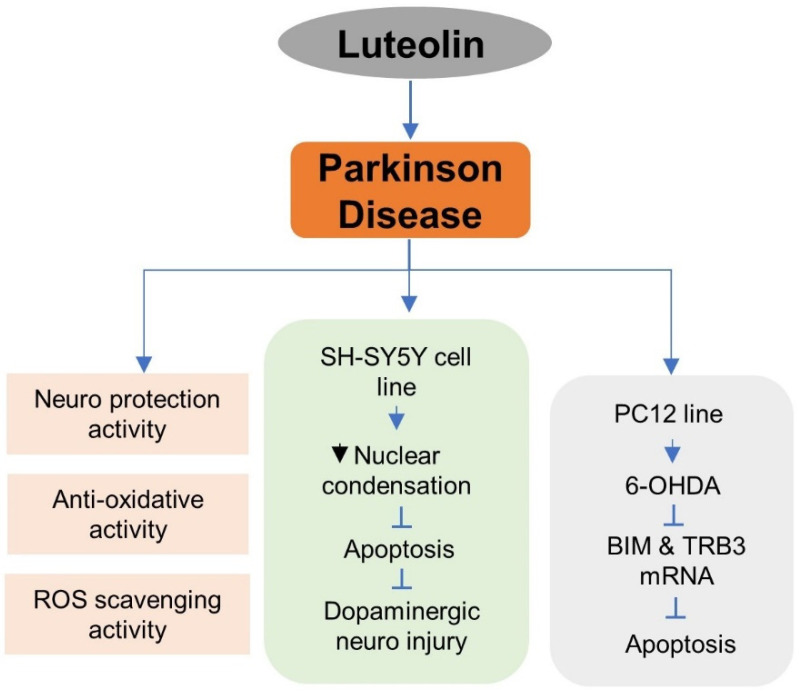
Protective role of luteolin in Parkinson’s treatment.

The superoxide dismutase activity is enhanced due to LUT, which helps to decompose OH-mediated lipid peroxidation. Luteolin suppresses the higher expression of Cyclin-dependent kinase-5 (Cdk5) and p35 due to oxidation stress. Thus it proves its effectiveness in influencing the extracellular signal-regulated kinase 1/2 (Erk1/2)- and dynamin-related protein 1 (Drp 1)-dependent survival pathways [99] (Figure 6).

### 5.7. Luteolin in Cardiac Health

The luteolin compound in various plant sources has many beneficial properties in favor of a healthy and disease-preventive lifestyle for humans. It possesses the property of managing heart ailments such as myocardial infarction. Cardiovascular diseases (CVDs) can result from various factors such as unhealthy lifestyle, unbalanced diet, and sedentary lifestyle. They can be prevented by eating healthy food and having an active lifestyle. The luteolin molecule helps to reduce the risk of myocardial infarction, as its inclusion in food may help in reducing CVD. In a study involving myocardial ischemia/reperfusion (I/R) (MIRM) rats, the luteolin compound treatment reduced the damage to the heart valves by reducing the Src homology 2 domain-containing protein tyrosine phosphatase 1 (SHP-1) regulation and upregulating the STAT3 pathway, resulting in decreased inflammatory response [100]. 

The reduced damage to heart muscles is due to the luteolin treatment, as it reduces the cytokine level in the serum of the treated animal models. In addition, luteolin helps in balancing the Siti1/NLRP3/NF-κB pathway, as it was affected in the MIRM rats [101]. The administration of luteolin to MIRM rats also proved to increase the sarcoplasmic/endoplasmic reticulum Ca^2+^-ATPase (SERCA) protein level by SUMOylation, and the expression of the SERCA was modulated by the Sp1 transcription factor, which has positive regulation over the *SERCA* gene. This process helped recover the heart tissue injury caused by myocardial infarction compared to the control [102,103]. 

The anti-apoptosis property is important in preventing heart tissue damage. In one study, administering luteolin reduced apoptosis in the simulated ischemia/reperfusion (sI/R) model by upregulating the AKT signaling [104]. Cardiac wellness is improved by luteolin, as it helps in preventing cardiac abnormalities such as contractile dysfunction and Ca^2+^ transport, which reduce in the failing cardiomyocytes and are prevented by upregulating the *SERCA2a* gene [105]. The importance of the SERCA proteins in cardiac health is evident. Luteolin helps to upregulate its expression by activating the p38 MAPK pathway in the sI/R rat models and in the cardiomyocytes [106]. The cardiac protective effect of the luteolin molecule is proved in animal models and cell lines, implying its possible effectiveness in humans. Its intake as a dietary compound could play a possible preventive role against life-threatening heart disease. 

### 5.8. Luteolin in Obesity Treatment

Another lifestyle disorder is obesity, which is the high accumulation of fats. It leads to many non-communicable disorders such as cardiac arrest and diabetes, musculoskeletal disorders such osteoarthritis, and cancers such as colon, ovarian, breast, liver, prostate, kidney, and gallbladder [107]. Luteolin, through dietary supplements, has proved to manage obesity. A study on rat models showed that luteolin supplementation decreased adipokine/cytokine dysregulation and macrophage infiltration by modifying the Toll-like receptor (TLR) signaling pathway [108]. In another study, the luteolin compound helped in overcoming diet-induced obesity and also increased the metabolomic rates by activating the AMPK/PGC1α pathway [109]. The luteolin compound helps fight obesity by acting against adipocyte differentiation by regulating the TF peroxisome proliferator-activated receptor γ (PPARγ) [110]. 

The obesity studies involving C57BL/6N mice showed that artichoke leaves (AR), having luteolin, helped in reducing the obesity-related complications in mice when given along with a high-fat diet [111]. The obesity adipocyte inflammation observed on administering luteolin reduces inflammation by reducing the proinflammatory mediators in macrophages such as tumor necrosis factor-α (TNFα), monocyte chemoattractant protein (MCP-1), and NO, while co-cultivating with 3T3-L1 adipocytes and RAW264 macrophages. This is evidenced by the activity of luteolin in reducing the inflammation in the adipose tissue [112]. In another study involving diet-induced obesity mice, luteolin was involved in the regulation of cholesterol efflux genes such as liver X receptor α (*LXR-α*), scavenger receptor class B member 1 (*SRB1*), and ATP-binding cassette transporter G1 (*ABCG1*). It showed that luteolin reduces cholesterol by regulating the various genes involved in the cholesterol efflux pathway [113]. Thus, the luteolin compound helps in managing obesity by acting on various cellular mechanisms and helps manage and control obesity in model animals, which could be translated into treating humans.

## 6. Cytotoxic Studies

The phytocompounds used in managing or treating a particular disease or its related symptoms must be in a precise dose, so that other cellular functions are not affected by the compound administration. Thus, toxicity studies help provide crucial information for the compounds used in the study. The LUT compound toxicity was studied by treating human retinal microvascular endothelial cells (HRMECs) against the anti-angiogenic effect. The treatment with 10 μM of LUT had no toxic effect but increasing the concentration up to 100 μM affected the cells [114,115]. In another study, LUT treated with human lymphoblastoid TK6 cells showed cytotoxic activity at 24 h with a minimal lethal dose concentration of 2.5 μM. In addition, DNA damage was observed at the concentrations of 5 and 10 µM, measured by the alkaline comet assay and the γH2A.X protein level [116]. 

In a study involving *Verbena officinalis*, a traditional medicinal herb containing luteolin 3′-methyl ether 7-glucuronosyl-(1-2)-glucuronide, resulted in prenatal toxicity when administered in high doses during the gestation period in female Sprague Dawley rats [117]. In another study, 100 µg/ml of LUT caused DNA damage in Vero cells and lymphocytes [118]. The toxicity studies emphasize the safe usage of LUT in therapeutic treatments, as the higher dosage may cause side effects. Therefore, intense studies exploring the toxicity of the luteolin phytocompound will give more insight into the effective concentration of its doses for disease treatments.

## 7. Clinical Trials

Clinical studies for the compound LUT have been carried out with various objectives. According to the clinical trials website, 18 entries were found to be involved in using the LUT compound. Among them, three studies are in starting phase, seven studies have been completed, and two were terminated. A clinical study to treat the olfactory dysfunction of Severe Acute Respiratory Syndrome Coronavirus 2 (SARS-CoV-2)-affected persons showed that the administration of palmitoylethanolamide (PEA) and luteolin helped in the recovery of olfactory functions [119]. In another randomized clinical trial in hepatocellular carcinoma (HCC) patients, a standard transcatheter arterial chemoembolization (TACE) therapy was synergized with a traditional Chinese medicine Fuzheng Jiedu Xiaoji formulation (FZJDXJ) that constitutes several phytocompounds, including luteolin. The results showed that the synergistic application of LUT resulted in the prolongation of one-year overall survival (OS) and progression-free survival (PFS) cases in the trials, as the anti-cancer activity was proven in animal models by their role in influencing the AKT/CyclinD1/p21/p27 pathway [120]. 

Another clinical study involved LUT treatment of delirium, a condition of cognition and awareness disorder. Post-operative delirium conditions were prevented when subjects were administrated with 700 mg of co-ultra-micronized palmitoylethanolamide (PEA) + 70 mg luteolin [121]. The product Altilix^®^ contains LUT as one of the components used to treat cardiovascular and liver function in metabolic syndrome. The results of one study showed that intake of Altilix^®^ supplementation helps to improve liver and cardiovascular functions [122]. In another study, LUT enhanced exercise performance by increasing oxygen extraction in muscle and brain oxygenation in low and high doses [123]. The efficiency of LUT in treating autism spectrum disorders (ASD) was studied in a control clinical study in which results showed adaptive functioning improvement among the subjects. This proves the effectiveness of the LUT compound in treating complex disorders such as autism [124]. The clinical trials using LUT compounds prove its effectiveness in managing and treating several diseases and health issues. Future studies will further explore the therapeutic benefits of luteolin against several diseases.

## 8. Conclusions

The plant-derived phytocompound LUT is present in most plants on earth. Plants containing the LUT compound have been used in various traditional medicines. The advent of modern analytical techniques highlighted the occurrence of LUT in plants as an important secondary metabolite in different cellular responses. Meanwhile, LUT is currently being explored for its beneficial activity in treating various human ailments. It has been made clear that the plant-obtained compound is a potential candidate in the treatment of various diseases, as it possesses the properties of anti-cancer, anti-inflammatory, anti-diabetic, and antioxidative effects. These beneficial activities of LUT have been proven and validated in multiple studies.

From a future perspective, the synergistic application of LUT with other natural or synthetic drugs in chemopreventive studies could be carried out, as this approach will be more effective in controlling and managing diseases. In addition, the bioavailability of LUT can be enhanced by nanoformulation using nanotechnology, which will increase its efficacy in administration to humans and also caters to specific and efficient disease management. To make this possible, comprehensive studies should be carried out to unravel the role of LUT in treating several other diseases at a molecular level and explore the exact mechanism behind its beneficial activity. This paves the way for different strategies to employ in studies aimed at improving the well-being of humankind.

## Data Availability

Not applicable.
